# Validation of the Hologic Aptima Unisex and Multitest Specimen Collection Kits Used for Endocervical and Male Urethral Swab Specimens (Aptima Swabs) for Collection of Samples from SARS-CoV-2-Infected Patients

**DOI:** 10.1128/JCM.00753-20

**Published:** 2020-07-23

**Authors:** E. Avaniss-Aghajani, A. Sarkissian, F. Fernando, A. Avaniss-Aghajani

**Affiliations:** a Primex Clinical Laboratories, Van Nuys, California, USA; b La Cañada High School, La Cañada Flintridge, California, USA; Boston Children's Hospital

**Keywords:** SARS-CoV-2, COVID-19, Aptima swabs, collection device

## LETTER

Recent events have seen the rise of severe acute respiratory syndrome coronavirus 2 (SARS-CoV-2) all across the world affecting the lives and economies of every nation. Originally identified in Wuhan, China, the virus has spread at an incredible rate to become a pandemic. Causing flu-like symptoms and severe respiratory problems, it has been shown to have a high fatality rate worldwide (https://www.who.int/emergencies/diseases/novel-coronavirus-2019). Large-volume testing has been a necessary tool for combating the spread of the virus and identification of infected individuals for focused care and isolation. A combination of the virus’ high infection rate, unprecedented volume testing need, and lack of world preparation has left testing health care providers without sufficient supplies in order to test samples at an efficient rate. Most notably, the flocked swabs and universal transport media (UTM) that are the primary collections tools for virtually all real-time PCR (RT-PCR) tests available for detection of SARS-CoV-2 have been on severe shortage, hampering testing all across United States.

To combat this shortage, we validated the Hologic Aptima unisex and multitest specimen collection devices. A total of 35 patients were swabbed simultaneously with two different collection swabs. The flocked swab was used in conjunction with UTM as the control for collection of nasopharyngeal samples. The Aptima unisex blue swab was used for collection of nasopharyngeal samples, and the Aptima multitest pink swab was used for the collection of nasal samples. Both were transported using Aptima transport media. One nostril was swabbed with the flocked swab and the other with the Aptima swab. The flocked swab/UTM samples were refrigerated, and the Aptima samples were kept at room temperature. A 100-μl volume of each sample was extracted using a Roche Magna Pure 96 DNA and viral nucleic acid (NA) small-volume kit with a final elution volume of 100 μl. A 5-μl volume of the extract was used for the RT-PCR using a Thermo Fisher TaqPath coronavirus disease 2019 (COVID-19) combo kit and a QuantStudio 12K Flex instrument. There were 16 positive and 19 negative samples in this group. In all positive cases except one, the levels of threshold cycle (*C_T_*) signal for the viral targets were significantly lower, indicating higher analytical sensitivity. The results are summarized in Table 1.

**TABLE 1:** Summary of *C_T_* results for the 3 SARS-CoV-2 target genes (N, S, and Orf-1ab) from 16 positive samples simultaneously swabbed with both Aptima swab/transport media and flocked swab/UTM collected and tested over 5 days^*a*^

Target gene	Swab	*C_T_*			95% CI	*P* value^*b*^
		Mean	Median	Range		
N	Aptima	27.3	29	18.6	(24.6,30.1)	0.011
	UTM	31.5	31.3	23.2	(28.1,35.0)	

ORF-1ab	Aptima	27.9	28.9	12.9	(25.9,29.8)	0.015
	UTM	31.4	31.6	23.7	(28.0,34.7)	

S	Aptima	27.8	28.4	11.8	(25.5,30.2)	0.014
	UTM	31.8	31.7	24.4	(28.0,35.5)	

a Aptima, Aptima swab/transport media; CI, confidence interval; UTM, flocked swab/universal transport media.

b UTM-Aptima.

All results were found to match between the two sets of swabs from the patients except three, which were positive in Aptima testing and negative with the flocked swabs. Two of these patients had tested positive previously at our laboratory with flocked swabs/UTM samples. The third patient sample was clearly positive with the Aptima swab/transport media only but was negative with the flocked swab. This was a new patient; therefore, the physician was notified of the results.

The Aptima swab and transport media were also tested for linearity and limit of detection (LoD) with quantitated genomic RNA from BEI Resources with an LoD of at least 250 genome equivalents/ml, which was comparable to the flocked swab/UTM LoD. Aptima samples were further tested for stability at room temperature. A total of 5 positive and 2 negative samples were tested in triplicate as the baseline, stored at room temperature for 4 days, and tested again in duplicate. The differences in the *C_T_* means before and after storage for the N protein, Orf-1ab, and S protein gene targets were 0.1, 0.3, and 0.5, respectively, proving the stability of the results after 4 days at room temperature.

Our results indicate that the Aptima swab collection and transport device appears to be an appropriate system for collection of samples from SARS-CoV-2-infected patients. It seemed to perform better than the flocked swabs/UTM in our tests. We believe this is due to the preservatives present in the Aptima swab solution (1) that prevent the breakdown of the viral RNA. Another advantage of the swab is that it might render the virus inactive due to a high concentration of detergents (2, 3) and might make the sample safer to handle. Finally, the swabs were field tested at room temperature, alleviating the need for refrigeration after collection and during transport.
